# The subjective health of adults in Germany

**DOI:** 10.17886/RKI-GBE-2018-073

**Published:** 2018-06-27

**Authors:** Thomas Lampert, Claudia Schmidtke, Lea-Sophie Borgmann, Christina Poethko-Müller, Benjamin Kuntz

**Affiliations:** Robert Koch Institute, Department of Epidemiology and Health Monitoring

**Keywords:** SUBJECTIVE HEALTH, GENERAL HEALTH, HEALTH DISPARITIES, HEALTH MONITORING

## Abstract

The term ‘subjective health’ reflects not only existing illnesses and health complaints, but particularly emphasizes the personal well-being. Studies often collect data on subjective health by asking participants to provide self-assessments of their general state of health. This was also the case with GEDA 2014/2015-EHIS, which employed the internationally renowned Minimum European Health Module (MEHM) as part of the study. Its results demonstrate that 68.2% of adults in Germany rate their general health as very good or good, with the remaining 31.8% rating it as fair, poor or very poor. The proportion of women who rate their general health as very good or good is slightly lower than the proportion of men who do so (66.6% compared to 69.9%). With increasing age, women and men view the condition of their general health as worsening. The study also identified educational differences which showed that men and women with low levels of education tend to rate their health worse compared to self-assessments provided by women and men with higher levels of education, and in some cases also regional differences.

## Introduction

Subjective health plays an integral role in numerous population-based health studies [1]. On the one hand, it includes existing illnesses and complaints, however, it also particularly takes people’s personal well-being into account. As such, a measurable relationship exists between objective and subjective health, however, these factors are not completely identical [2]. Subjective health is often measured with the self-assessment of general health, which has been shown to be a reliable predictor of future health service utilization and mortality [3-6]. Furthermore, a correlation exists between the incidence of chronic diseases and functional impairments over time, and the ratings that a person has previously provided of their health [7, 8]. Associations also exist between health-related behaviour and the motivation that people have to adopt a health-promoting lifestyle and to actively participate in society [9, 10]. Social differences in self-assessments of general health, such as those between educational and income groups, therefore, also provide indications of health disparities, which, in turn, are reflected in socially unequal distributions of diseases, complaints and health risks and the resulting need for care [11].

## Indicator

Data on subjective health was gathered for the GEDA 2014/2015-EHIS study using information provided by the respondents as part of a questionnaire that was either completed on paper or online. In accordance with World Health Organization (WHO) recommendations, respondents were asked, ‘How is your health in general?’ [12]. They were able to select one of five predefined options: ‘very good’, ‘good’, ‘fair’, ‘bad’ or ‘very bad’. This question forms part of the internationally renowned Minimum European Health Module (MEHM), which is often used in health surveys [13]. The GEDA studies that took place in 2009, 2010 and 2012 were conducted as telephone interviews and also used this questionnaire to collect data on subjective health [14]. The results presented in the following either encompass all five answer options, or focus on the respondents who assessed their health as very good or good.

**GEDA 2014/2015-EHIS**

**Data holder:** Robert Koch Institute

**Aims:** To provide reliable information about the population’s health status, health-related behaviour and health care in Germany, with the possibility of a European comparison

**Method:** Questionnaires completed on paper or online

**Population:** People aged 18 years and above with permanent residency in Germany

**Sampling:** Registry office sample; randomly selected individuals from 301 communities in Germany were invited to participate

**Participants:** 24,016 people (13,144 women; 10,872 men)

**Response rate:** 26.9%

**Study period:** November 2014 - July 2015

**More information in German is available at**

www.geda-studie.de

The following analyses are based on data from 23,906 participating individuals aged 18 years or older (13,077 women, 10,829 men) who provided valid information about the general state of their health. Calculations were carried out using a weighting factor that corrected the sample for deviations from the population structure (on 31 December 2014) in terms of gender, age, municipality type and level of education. The municipality type reflects the degree of urbanisation in a particular area and corresponds to the way in which urbanisation is distributed throughout Germany. The International Standard Classification of Education (ISCED) was used to classify the participants’ educational and occupational qualifications [15]. A statistically significant difference between groups was assumed to have been demonstrated if p-values were lower than 0.05.

A detailed description of the methodology employed for GEDA 2014/2015-EHIS can be found in Lange et al. 2017 [16] as well as in the article German Health Update: New data for Germany and Europe in issue 1/2017 of the Journal of Health Monitoring [17].

## Results and discussion

According to the data collected for the GEDA 2014/2015-EHIS survey, 68.2% of adults in Germany rate their general health as very good or good. However, the proportion of women who do so is at 66.6% somewhat lower (Table 1) than men (69.9%, Table 2). Differences also exist between age groups: 18- to 29-year-olds most frequently rate their general health as very good or good (85.0%). Among people aged 65 or above, this is the case with just 47.5%. Moreover, a comparison of the various age groups demonstrates that the differences between women and men only exist in the youngest age group: 80.4% of women aged between 18 and 29 years rate their health as very good or good, compared to 89.3% of men in the same age group. Although the proportion of women in other age groups who rate their general health as very good or good is also slightly lower than men in the same age groups, these differences are not statistically significant.

Significant differences were identified between educational groups (Table 1 and Table 2): a total of 77.9% of people with a high level of education rate their general health as very good or good compared to just 56.5% of those with a low level of education. In addition, 68.4% of people with a medium level of education describe their general health as very good or good. This educational gradient – which disadvantages people with low levels of education – is equally evident among women and men, however, educational differences are more pronounced in some age groups than others.

**Figure 1:**
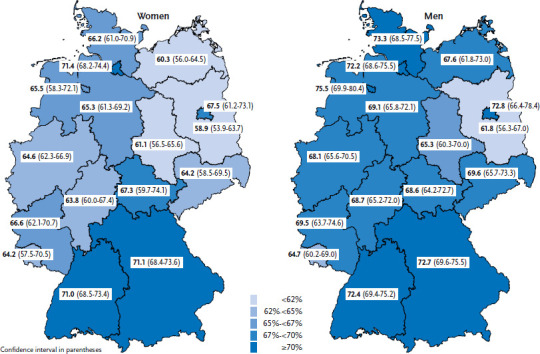
The proportion of women and men with very good or good general health according to federal state (n=13,077 women, 10,829 men) Source: GEDA 2014/2015-EHIS

Lastly, regional differences were identified (Figure 1). The proportion of people who rate their general health as very good or good is highest in Bavaria and Hamburg (both 71.8%) and Baden-Württemberg (71.7%). In Brandenburg, Saxony-Anhalt and Mecklenburg-Vorpommern, this proportion is lowest at 60.3%, 63.2% and 63.9%, respectively. These regional differences were identified among both women and men.

Compared to the GEDA studies that were conducted in 2009, 2010 and 2012, the proportion of women and men who rate their general health as very good or good is slightly lower in the GEDA 2014/2015-EHIS survey. However, it should be noted that previous GEDA studies were conducted as telephone interviews. The literature clearly demonstrates that survey methods have an impact on results (‘mode effect’). In this case, participants would tend to provide a more favourable assessment of their own health when questioned using telephone surveys than, for example, written surveys [18, 19]. Nevertheless, the results of the GEDA studies consistently show that the majority of adults in Germany view their own general health as very good or good. However, people who are seriously ill, impaired or in hospital may have been less likely to participate in the study. The differences in age, gender and education point on existing potential for improving the health status of the population. In order to find concrete approaches to disease prevention, health promotion and health care measures, further analyses of specific diseases and risk factors are required.

## Key statements

- Two-thirds of adults in Germany describe their general health as very good or good.
- The proportion of people who rate their health as very good or good decreases with age.
- People with lower levels of education rate their health worse compared to the self-assessments provided by people with higher levels of education.
- People living in Bavaria, Baden-Württemberg and Hamburg provide the most positive self-assessments of their health; the lowest ratings came from Brandenburg, Saxony-Anhalt and Mecklenburg-Vorpommern.

## Figures and Tables

**Table 1:** Self-assessed general health among women according to age and educational level (n=13,077) Source: GEDA 2014/2015-EHIS

Women	Very good		Good		Fair		Bad		Very bad	
	%	(95% CI)	%	(95% CI)	%	(95% CI)	%	(95% CI)	%	(95% CI)
**Women (total)**	**13.9**	**(13.2-14.7)**	**52.7**	**(51.6-53.7)**	**27.9**	**(26.9-28.9)**	**4.8**	**(4.3-5.3)**	**0.7**	**(0.6-1.0)**
**18-29 Years**	24.2	(22.0-26.6)	56.2	(53.7-58.7)	17.1	(15.1-19.3)	2.4	(1.6-3.5)	0.1	(0.0-0.4)
Low education	20.6	(16.0-26.1)	49.6	(43.6-55.5)	25.0	(19.9-30.8)	4.4	(2.2-8.5)	0.5	(0.1-1.8)
Medium education	23.4	(21.0-26.1)	58.7	(55.3-61.9)	15.9	(13.5-18.6)	2.0	(1.2-3.4)	-	-
High education	33.0	(28.3-38.0)	56.8	(51.5-61.9)	9.6	(7.0-13.0)	0.7	(0.2-2.0)	-	-
**30-44 Years**	19.9	(18.3-21.5)	60.4	(58.2-62.5)	17.4	(15.8-19.2)	2.0	(1.4-2.7)	0.4	(0.2-0.7)
Low education	15.1	(10.6-21.1)	55.2	(48.6-61.7)	24.0	(18.5-30.7)	4.4	(2.5-7.6)	1.2	(0.4-3.8)
Medium education	17.0	(15.0-19.2)	62.0	(59.1-64.9)	18.7	(16.6-21.0)	2.0	(1.3-3.0)	0.2	(0.1-0.7)
High education	29.4	(26.1-33.0)	59.2	(55.5-62.7)	10.8	(8.9-12.9)	0.5	(0.2-1.2)	0.1	(0.0-1.0)
**45-64 Years**	12.7	(11.6-13.9)	54.9	(53.1-56.6)	27.1	(25.6-28.7)	4.7	(4.1-5.5)	0.5	(0.3-0.9)
Low education	9.0	(6.8-11.9)	47.0	(42.6-51.5)	34.9	(30.8-39.2)	7.3	(5.4-9.9)	1.8	(0.8-3.9)
Medium education	12.2	(10.8-13.7)	56.2	(53.9-58.4)	26.6	(24.7-28.7)	4.7	(3.9-5.7)	0.4	(0.2-0.7)
High education	17.9	(15.9-20.2)	57.7	(54.5-60.7)	21.8	(19.5-24.2)	2.7	(1.9-3.8)	-	-
**≥65 Years**	4.4	(3.6-5.5)	41.3	(38.9-43.6)	44.0	(41.7-46.3)	8.6	(7.4-10.0)	1.7	(1.2-2.5)
Low education	4.0	(2.8-5.7)	34.5	(30.9-38.2)	49.0	(45.5-52.5)	10.3	(8.4-12.5)	2.3	(1.3-3.9)
Medium education	4.4	(3.2-6.0)	45.4	(42.3-48.5)	41.3	(38.1-44.6)	7.4	(5.7-9.5)	1.5	(0.9-2.7)
High education	6.6	(4.7-9.1)	50.6	(45.7-55.6)	34.4	(30.1-39.1)	7.8	(4.8-12.6)	0.5	(0.1-2.2)
**Total (women and men)**	**14.8**	**(14.2-15.4)**	**53.4**	**(52.6-54.2)**	**26.3**	**(25.6-26.9)**	**4.8**	**(4.5-5.2)**	**0.7**	**(0.6-0.9)**

CI=confidence interval

**Table 2:** Self-assessed general health among men according to age and educational level (n=10,829) Source: GEDA 2014/2015-EHIS

Men	Very good		Good		Fair		Bad		Very bad	
	%	(95% CI)	%	(95% CI)	%	(95% CI)	%	(95% CI)	%	(95% CI)
**Men (total)**	**15.7**	**(14.8-16.7)**	**54.2**	**(53.0-55.3)**	**24.5**	**(23.6-25.6)**	**4.8**	**(4.4-5.3)**	**0.7**	**(0.6-1.0)**
**18-29 Years**	34.2	(31.2-37.3)	55.1	(51.8-58.4)	9.7	(8.0-11.8)	0.9	(0.5-1.7)	0.1	(0.0-0.4)
Low education	28.9	(22.9-35.7)	53.4	(46.3-60.3)	16.2	(11.6-22.1)	1.6	(0.6-4.3)	-	-
Medium education	33.0	(29.3-36.9)	58.3	(54.1-62.4)	8.1	(6.2-10.5)	0.5	(0.2-1.0)	0.1	(0.0-0.7)
High education	49.0	(42.4-55.6)	44.8	(38.6-51.1)	4.8	(2.7-8.4)	1.4	(0.2-8.9)	-	-
**30-44 Years**	20.9	(18.8-23.2)	60.2	(57.6-62.7)	16.0	(14.2-18.1)	2.5	(1.8-3.5)	0.4	(0.2-0.9)
Low education	16.4	(10.9-23.9)	49.5	(40.8-58.2)	28.8	(21.4-37.5)	4.4	(2.1-9.1)	0.9	(0.2-4.0)
Medium education	18.0	(15.4-20.9)	61.4	(57.9-64.8)	17.1	(14.6-20.1)	3.0	(2.0-4.5)	0.4	(0.1-1.4)
High education	27.7	(24.3-31.3)	62.7	(58.8-66.5)	8.8	(6.9-11.1)	0.8	(0.4-1.7)	0.1	(0.0-0.5)
**45-64 Years**	10.5	(9.5-11.7)	55.1	(53.4-56.8)	27.6	(26.0-29.1)	5.9	(5.1-6.8)	0.9	(0.6-1.4)
Low education	6.4	(4.2-9.5)	49.3	(44.1-54.6)	34.1	(29.3-39.1)	7.6	(5.3-10.7)	2.7	(1.3-5.3)
Medium education	8.5	(7.2-10.1)	52.3	(49.9-54.7)	31.3	(29.0-33.7)	7.2	(5.9-8.6)	0.7	(0.4-1.5)
High education	15.7	(13.8-17.8)	62.3	(59.7-64.9)	18.4	(16.4-20.6)	3.0	(2.2-4.1)	0.6	(0.3-1.2)
**≥65 Years**	4.1	(3.4-5.0)	45.7	(43.5-48.0)	40.2	(37.8-42.6)	8.6	(7.3-10.0)	1.4	(1.0-2.1)
Low education	2.3	(1.2-4.2)	42.7	(38.0-47.5)	44.4	(39.7-49.3)	8.8	(6.3-12.1)	1.9	(1.0-3.6)
Medium education	3.4	(2.5-4.7)	44.5	(41.2-47.8)	41.0	(37.8-44.2)	9.6	(7.9-11.7)	1.5	(0.9-2.7)
High education	6.4	(5.1-8.1)	49.4	(46.0-52.9)	36.5	(33.0-40.2)	6.7	(5.1-8.7)	1.0	(0.5-1.9)
**Total (women and men)**	**14.8**	**(14.2-15.4)**	**53.4**	**(52.6-54.2)**	**26.3**	**(25.6-26.9)**	**4.8**	**(4.5-5.2)**	**0.7**	**(0.6-0.9)**

CI=confidence interval
